# Criteria for Reporting the Development and Evaluation of Complex Interventions in healthcare: revised guideline (CReDECI 2)

**DOI:** 10.1186/s13063-015-0709-y

**Published:** 2015-05-03

**Authors:** Ralph Möhler, Sascha Köpke, Gabriele Meyer

**Affiliations:** School of Nursing Science, Faculty of Health, Witten/Herdecke University, Stockumer Straße 12, D-58453 Witten, Germany; Institute of Health and Nursing Science, Medical Faculty, Martin-Luther-University Halle-Wittenberg, Magdeburger Straße 8, D-06112 Halle (Saale), Germany; Nursing Research Unit, Institute of Social Medicine, University of Lübeck, Ratzeburger Allee 160, D-23538 Lübeck, Germany

**Keywords:** complex interventions, consensus process, reporting guideline, research design

## Abstract

**Background:**

Many healthcare interventions are of complex nature, consisting of several interacting components. Complex interventions are often described inadequately. A reporting guideline for complex interventions was published in 2012 (Criteria for Reporting the Development and Evaluation of Complex Interventions in healthcare, CReDECI) and was recently checked for its practicability. The reporting guideline was developed following the recommendations of the EQUATOR network but excluding a formal consensus process. Therefore, a consensus process was initiated, to revise the reporting guideline.

**Methods:**

We used a three-phase consensus process consisting of (1) a web-based feedback survey on the published reporting guideline, (2) a face-to-face consensus conference, and (3) a final online review and feedback round to create the revised CReDECI. The consensus process was organized and conducted via the REFLECTION network.

**Results:**

A total of 45 attendees from 16 European countries took part in the face-to-face consensus conference. The revised reporting guideline (CReDECI 2) comprises 13 items on three stages: development, feasibility and piloting, and evaluation of a complex intervention. Each item is illustrated by an explanation and an example. In contrast with most of the available reporting guidelines, CReDECI 2 does not focus on a specific study design, to reflect the use of different qualitative and quantitative designs and methods in the development and evaluation of complex interventions.

**Conclusions:**

CReDECI 2 is a formally consented reporting guideline aiming to improve the reporting quality of the development and evaluation stages of complex interventions in healthcare. Since the guideline does not focus on a specific study design, design-specific reporting guidelines may additionally be used.

**Electronic supplementary material:**

The online version of this article (doi:10.1186/s13063-015-0709-y) contains supplementary material, which is available to authorized users.

## Background

The quality of reports of empirical studies has become an important issue in the last two decades. In 1996, the first CONSORT statement was published to improve the reporting quality of randomized controlled trials (RCTs) [1]. In the following years, reporting guidelines for various study designs or specific types of study design, such as cluster RCTs or pragmatic trials, have been published. In 2006, the EQUATOR network was established, which aimed at improving the reliability and value of published health research literature by promoting transparent and accurate reporting and wider use of robust reporting guidelines [2]. At the time of writing, more than 200 reporting guidelines are listed in the EQUATOR database [3].

Another topic that has been discussed intensively in recent years reflects the challenges of developing and evaluating complex healthcare interventions. Complex interventions consist of several components, which can act either independently or interdependently [4]. Characteristics that make interventions complex are different professions or different organizational levels targeted by the intervention (context of the intervention) and/or a need to tailor the intervention for specific settings (flexibility of the intervention) [4]. Most nonpharmacological, behavioral change and educational interventions are likely to be complex interventions [4,5]. In 2000, the British Medical Research Council (MRC) developed a framework for the development and evaluation of complex interventions offering methodological guidance [6]. An intensive discussion on methodological aspects of this framework led to an updated version in 2008 [4]. The MRC framework offered methodological recommendations for the development, feasibility and piloting, evaluation, and long-term implementation of complex interventions. Additionally, the framework recommended comprehensive reporting of all stages within the research process, adhering to the relevant reporting guidelines [4]. A specific reporting guideline, however, was lacking until 2012. Therefore, we developed the Criteria for Reporting the Development and Evaluation of Complex Interventions in healthcare (CReDECI) [7]. In contrast with most reporting guidelines, CReDECI does not offer criteria for a specific study design, but on the process of development, piloting, and evaluation of complex interventions. Several reporting guidelines or criteria sets are available, which cover relevant aspects for comprehensive reporting of complex interventions (Table 1). However, the majority of these guidelines are CONSORT extensions and have been developed solely to be used for the publication of RCTs. For complex interventions, comprehensive reporting of all stages of the research process is required (as recommended by the MRC framework [4]). For example, the development of complex interventions is often reported in an extra publication or in the study protocol of the evaluation study, where CONSORT [8] is not applicable.

**Table 1:** **Available reporting guidelines covering aspects of complex interventions**

**Title**	**Content**
CONSORT extensions for cluster randomized controlled trials [9], nonpharmacological interventions [10], pragmatic trials [11], social and psychological intervention trials (under development) [12]	Comprise some aspects also relevant in clinical trials of complex interventions. Not specific for complex interventions; exclusively focused on clinical trials (randomized controlled trials)
Workgroup for Intervention Development and Evaluation Research recommendations [13]	Set of criteria focusing on a comprehensive description of behavior change interventions to improve replicability
Template for Intervention Description and Replication [14]	Focuses on a comprehensive description of interventions to improve replicability. Developed as an extension of the CONSORT 2010 statement (item 5) [8] and the SPIRIT 2013 statement (item 11) [15]
Grant *et al.* [16]	Set of criteria for reporting process evaluation in (cluster) randomized controlled trials of complex interventions

The first version of the CReDECI guideline comprised 16 items, covering the development, feasibility and piloting, and evaluation stages of complex interventions. CReDECI proved to be a feasible instrument [17]. The guideline was created adhering to the recommendations of the EQUATOR network, with the exception of a formal consensus process [18]. Therefore, we aimed to revise the CReDECI list based on a formal consensus conference.

## Methods

### Design and participants

The original CReDECI guideline was revised in three steps: (1) feedback on the first CReDECI guideline was collected via online questionnaires, (2) a face-to-face consensus conference was held to develop a draft revised guideline, and (3) the revised guideline was sent to all consensus conference attendees to reach a final consensus.

Participants for the consensus process were recruited via the REFLECTION network [19]. REFLECTION is a European research network, funded by research councils and academies from eight European countries under the umbrella of the European Science Foundation. REFLECTION aims to promote high quality research on complex nursing interventions and to share knowledge and expertise [19].

### Procedure

To collect feedback on aspects relevant for revising the reporting guideline, an online questionnaire was developed and piloted. Participants were invited to comment on all items of the original CReDECI guideline (including the explanations and examples). In addition, we asked for general feedback on the guideline, as well as comments on aspects omitted in the included stages (development, piloting, and evaluation of a complex intervention). Within the REFLECTION network, a specific group of 38 researchers was formed for the consensus process. The questionnaire was distributed via this group and was available online for six months (April 2013 to end of September 2013). All group members were invited to complete the questionnaire and two reminders were sent. Based on the online feedback, a draft revision of the guideline was prepared for discussion at the face-to-face consensus conference. This conference was held at the REFLECTION masterclass on methods of complex interventions in October 2013 in Nitra, Slovakia. The REFLECTION masterclass offers seminars and workshops on specific topics of complex interventions research. Participation is open to European healthcare researchers working in the field of complex interventions. In addition to members of the REFLECTION network consensus group, all the participants of the masterclass were invited to join the consensus conference.

A total of 45 participants took part in the consensus conference. The attendees were researchers in the field of nursing science, health services research, and health psychology from 16 European countries. Of the 45 attendees, 19 were professors, another 16 had a PhD, and nine had a master’s degree. Six attendees were also editors or members of editorial boards of nursing or healthcare journals. All attendees had experience in the development and evaluation of complex healthcare interventions and several had conducted or coordinated evaluation studies (partly as consortium members of multinational projects funded by the European Union).

The conference started with a brief presentation of the original CReDECI list and an introduction about the aim and methods of the consensus conference. Following this, attendees were divided into three groups of equal size to discuss a set of items in detail. Each group was provided with all the comments from the online round and the first draft revision of the group’s items. In each group, one author of the original CReDECI list (RM, SK, GM) moderated the discussions and collected the feedback. The group meetings lasted 90 minutes. Afterwards, the participants met again to discuss the changes proposed by the three groups and a final draft of the items with their explanations and examples was developed.

Based on the comments and changes from the consensus conference, a final draft was created by the authors and sent to all participants of both the consensus conference and the REFLECTION group (59 participants). After a further round of additional comments, the final revised guideline was approved.

Participants informally consented to take part in this study. No formal written informed consent was collected as all attendees volunteered to join the REFLECTION project as well as the workshop. All attendees were informed that they would be acknowledged in this publication (see acknowledgement).

The original CReDECI tool had been developed based on a systematic literature review of the methodological literature about the development and evaluation of complex interventions in healthcare [7]. Although mostly researchers from the nursing field were involved in the development of the tools, both CReDECI and CReDECI 2 have not been developed specifically for studies in nursing or any other discipline, but for generic use in all healthcare interventions (equally to the MRC framework [4]).

## Results

The CReDECI 2 list comprises 13 items for the stages development, piloting, and evaluation of a complex intervention (compared with 16 items in the original list). Four items were merged and one item was split. For each item, an explanation and an example is presented (an overview of the original and the revised criteria is presented in Additional file 1). In contrast with the first CReDECI guideline, the revised list comprises examples from real studies.

### The revised criteria list (CReDECI 2)

In the following, all items of CReDECI 2 are presented. A reference is given for each example; however, references within examples are not displayed, but indicated by [Ref.]. A checklist with all items but without explanations and examples is presented in Table 2.

**Table 2:** **CReDECI 2 checklist**

**Item**		**Reported on page or in publication**
**First stage: Development**		
1	Description of the intervention’s underlying theoretical basis	
2	Description of all intervention components, including the reasons for their selection as well as their aims / essential functions	
3	Illustration of any intended interactions between different components	
4	Description and consideration of the context’s characteristics in intervention modelling	
**Second stage: Feasibility and piloting**		
5	Description of the pilot test and its impact on the definite intervention	
**Third stage: Evaluation**		
6	Description of the control condition (comparator) and reasons for the selection	
7	Description of the strategy for delivering the intervention within the study context	
8	Description of all materials or tools used delivery the intervention	
9	Description of fidelity of the delivery process compared the study protocol	
10	Description of a process evaluation and its underlying theoretical basis	
11	Description of internal facilitators and barriers potentially influencing the delivery of the intervention as revealed by the process evaluation	
12	Description of external conditions or factors occurring during the study which might have influenced the delivery of the intervention or mode of action ( how it works)	
13	Description of costs or required resources for the delivery of the intervention	

### First stage: development

**Item 1**: Description of the intervention’s underlying theoretical basis.

**Explanation**: The theoretical basis of the intervention includes specific theories, theoretical positions, and frameworks guiding the development, design, and evaluation of the intervention, as well as, if available, empirical evidence, that is, from studies conducted in different settings or countries.

**Example** [20]: “*We developed a multifaceted intervention comprising care plans for both the practices and the patients (…). By exploring practitioners’ and patients’ views (…)*, *we determined the duration and intensity of training within the practices and the frequency of patient recall. (…) cognitive theory* [Ref] *was the main psychological theory used to develop the training in behavior change, design the booklet for intervention patients (Ref), and inform the development of tailored plans for patient care. According to this theory, building patients’ or and facilitating patients in setting goals and making action plans are central to the optimal management of chronic disease*”.

**Item 2**: Description of all intervention components, including the reasons for their selection as well as their aims/essential functions.

**Explanation**: Complex interventions are composed of several interacting components. The description of the components includes the reasons for selecting a specific component (for example, experience of or evidence on the suitability of the component to achieve the intended change process), and the characteristics of the components (for example, the components’ target population, duration, sequence, and frequency of delivery). Also, the description of the aim or essential function of the components rather than the content in detail is needed, for example, the content of an education program might differ more between various countries than the aim or essential function. A graphical presentation of the components might be useful [21].

(see Table 3).

**Table 3:** **Components of the intervention (excerpt)** [22]

***Intervention***	***Description***	***Basis of rationale***
*Declaration*	• *Declaration confirming the nursing home’s dedication to the intervention’s objectives, i.e. the avoidance of physical restraints signed by head nurses and/or directors of each nursing home*	• *Proven strategy in previous studies* ^*(Ref)*^
*Structured 90-minute information program for all nursing staff*	• *Definition of physical restraints*	• *Cochrane review* ^*(Ref)*^
	• *Desired and unwanted effects of physical restraints*	• *Theory of planned behavior* ^*(Ref)*^
	• *Legal aspects of physical restraint use*	• *Acknowledging perceived barriers, current practice culture, and concerns and emotional responses of nursing staff by using different educational strategies, e.g. working with case vignettes and small group work* ^*(Ref)*^
	• *Guideline development and recommendations*	
	• *Nurses’ subjective attitudes and experiences*	
	• *Alternative approaches focusing on physical restraints avoidance as most important alternative*	• *…*
*External structured 1-day intensive training workshop for nominated key nurses from different nursing homes*	• *Advanced version of 90-min session*	• *Key nurses to support reduction of physical restraints* ^*(Ref)*^
	• *In-depth work with the guideline*	• *All aspects referred to in the above box*
	• *Exchange and discussion between nurses from different nursing homes*	
	• *Group presentation and discussion of individual barriers and facilitators of physical restraints reduction*	
	• *…*	

**Item 3**: Illustration of any intended interactions between different components.

**Explanation**: In some cases, different components are designed to support or to enhance the effect of other components. The description of all intended interactions between components is highly relevant. This could be supplemented by a graphical illustration.

**Example** [23]: “*A structured single information session of approximately 90 minutes will be provided for each cluster of the intervention group, so that at best all nurses will be informed. The information programme intends to sensitise nurses about the matter of physical restraints and the message of the guidance by addressing their subjective attitudes and experiences. By means of interactive training sequences nurses are motivated to discuss and develop alternative approaches. As supporting materials they receive a short version of the guidance and reminders like posters, pens, mugs, and note pads*”.

**Item 4**: Description and consideration of the context’s characteristics in intervention modelling.

**Explanation**: Considering the context of the intervention’s target setting is crucial for modelling of a complex intervention. Context conditions from different levels can be relevant: the macro level (for example, aspects of financing services, legal and political aspects, education of professionals), the meso level (for example, institutional or community-specific conditions) and the micro level (for example, teams, individuals, or local structures). The description of all aspects judged as relevant for modelling the intervention is of interest.

**Example** [20]: “*We recruited general practices from two different healthcare systems in Ireland. The Republic of Ireland has a mixed healthcare system and Northern Ireland is served by the UK National Health Service (…). Key features of healthcare systems in Northern Ireland and Republic of Ireland: (…)*”.

### Second stage: feasibility and piloting

**Item 5**: Description of the pilot test and its impact on the definite intervention.

**Explanation**: A pilot study aims to determine the feasibility, acceptability, and/or practicability of a complex intervention. Information on how the intervention was tested for procedural, clinical, and methodological uncertainties identified during the development process is crucial.

**Example** [24]: “*Problems were also identified through testing the process of plan production and delivery (…). Two substantial changes were made including the addition of a carer component and the introduction of manual checking procedures to ensure that all patient information provided in the plans is correct and all related secondary prevention advice is appropriate*”.

### Third stage: evaluation

**Item 6**: Description of the control condition (comparator) and reasons for the selection.

**Explanation**: Control conditions can comprise usual care (that is, standard care without any additional component), optimized usual care (that is, standard care with one or more additional components delivered by the research team), or an active control condition (that is, another intervention). The description of the characteristics of the control condition may cover information on professionals or services available for the target population, as well as information on differences in the control condition across study centers.

**Example** [20]: “*Usual care (…) in Northern Ireland involved a system for annual review of blood pressure, cholesterol concentration, smoking status, and prescribed drugs, in accordance with the criteria specified within the NHS general practitioner contract quality and outcomes framework for the management of coronary heart disease*”.

**Item 7**: Description of the strategy for delivering the intervention within the study context.

**Explanation**: A pre-planned strategy for delivering the intervention is crucial. This strategy aims at maintaining a standardized delivery of the intervention as far as possible (for example, in case of different study centers), but can also include methods to deal with local or personnel conditions and local tailoring of the intervention.

**Example** [25]: “*The training was standardized by using the same training materials: the trainers (the research general practitioner and research nurse in each center) adhered to a single training protocol, and training delivery was planned and rehearsed jointly by all trainers using role play and peer review (Ref)*”.

**Item 8**: Description of all materials or tools used for the delivery of the intervention.

**Explanation**: Interventions often comprise materials, for example, brochures, checklists, or flyers. Materials or tools can be components of the intervention by themselves (for example, patient’s diaries or short versions of guidelines) or a method to ensure the delivery or increase awareness towards the intervention (for example, posters or information sheets). These materials might impact the intervention effect; therefore information on their aims, content, format, and accessibility, is highly relevant.

**Example** [22]:

*‘Printed supportive material:*

- *Provision of the guideline’s 16-page short version for all nursing staff,*
- *Provision of the guideline’s 16-page short version for legal guardians and relatives focusing on legal aspects,*
- *Provision of a leporello-style flyer for relatives and other visitors with information about the project’s main objectives.*

*‘Other supportive material:*

- *Provision of posters with the intervention’s logo and slogan (‘Dare more freedom’),*
- *Provision of pencils and post-its with the intervention’s logo for all nurses attending the educational session*
- *Provision of mugs with the intervention’s logo for key nurses’.*

**Item 9**: Description of fidelity of the delivery process compared with the study protocol.

**Explanation**: Information on the actual delivery of the intervention and on any deviation from the study protocol during the study is of interest. If any deviation occurred, information on necessary adjustments of single components or the entire intervention is relevant. Adjustments may have been necessary in one or more centers or even the whole intervention group.

**Example** [26]: “*The training and intervention were delivered as planned for the general practitioners, practice nurses, and peer supporters in the protocol. All intervention and control practices implemented structured diabetes care as planned. All the practices and 28 out of the 29 peer supporters were followed up, though only 23 of the peer supporters were retained in their role. The main concern regarding the delivery and receipt of treatment, that is, the intervention, was the low attendance at the group meetings. Participants in the intervention group attended a mean of five peer support meetings, and 18% never attended a meeting and therefore had no exposure to the intervention. This was despite repeated phone calls from practice nurses and a call from the study manager to all nonattenders after the third round of meetings*”.

**Item 10**: Description of a process evaluation and its underlying theoretical basis.

**Explanation**: Process evaluation is a prerequisite in determining the success of the intervention’s delivery. Information on the theoretical basis, methods, and results of a process evaluation is relevant to understand the effects of the intervention. Process evaluation should be planned *a priori* and rely on an established framework, for example, Linnan and Steckler [27] or Grant [16].

**Example** [28]: “*We preplanned a process evaluation for our newly developed fall-prevention program (…). Because of the frailty of our population, we tried to assess as many variables as possible with simple questionnaires or registration forms. In addition, we performed short semistructured interviews among participants and instructors to gather information about their experiences and thoughts*”.

(see Table 4).

**Table 4:** **Table** **(excerpt)**

***Process Measures***	***Process Variables***
*1. Quality of delivery of the interventional components*	*1a. The part of each component and the complete intervention delivered by instructors; b. Satisfaction with delivery*
*2. Barriers and facilitators for delivery of interventional components*	*2. Reasons for diverging from, or applying (planned) components*
*3. Adherence to interventional components*	*3a. Number of sessions followed; b. Intervention components (partly) followed; c. Compliance to individual recommendations; d. Homework adherence*
*4. Barriers and facilitators for adherence to interventional components*	*4. Motivation for (lack of) attendance and compliance*
*5. Experience of participants and instructors with interventional components*	*5a. Perceived benefit; b. Strong and weak aspects of the interventional components (structure and content), and the total intervention”*

**Item 11**: Description of internal facilitators and barriers potentially influencing the delivery of the intervention as revealed by the process evaluation.

**Explanation**: The process evaluation may reveal internal facilitators or barriers identified within the study context, for example, resources, staff reluctance, or unforeseen staff turnover. It is important to describe facilitators or barriers from different perspectives, for example, participants, staff, research team.

**Example** [22]: “*The qualitative analysis of 40 in-depth interviews with nominated key nurses and head nurses identified important facilitators of and barriers to reducing prevalence of physical restraint use. Potential facilitators were supportive attitudes among head nurses; in-house quality circles with case discussion; counselling and education of relatives; and explicit and qualified information for judges, legal guardians, and physicians. Important barriers were negative experiences of nurses, concerns and uncertainties of relatives and legal guardians, and organizational problems (for example, staff fluctuation).*”

**Item 12**: Description of external conditions or factors occurring during the study that might have influenced the delivery of the intervention or mode of action (that is, how it works).

**Explanation**: External conditions or factors may be unforeseen changes in clinical practice that have been observed during the delivery of the intervention or during the study period. External conditions or factors might be the introduction of new guidelines, set up of policies or laws. or organizational changes.

**Example** [29]: “*In March 2004, the Committee on Safety of Medicines wrote to all doctors in the United Kingdom to advise against the prescription of risperidone and olanzapine in patients with dementia. The effect of this communication, which might have been expected to result in discontinuation of neuroleptics in a large number of participants in both arms of the trial, was only modest. Differences in the proportion of patients receiving neuroleptics at each review (…) between the groups were sustained over the year, and similarly affected by the ruling of the Committee on Safety of Medicines.*”

**Item 13**: Description of costs or required resources for the delivery of the intervention.

**Explanation**: Information on all expenses is needed, for example, personnel costs, material, or equipment.

**Example** [30]: “*The average direct cost of the intervention, including nurse time and psychiatrist supervision (but not the cost of nurse training or screening for depression), was £261.65 per patient. Patients who received the intervention also had slightly greater costs for healthcare than did those who had usual care (£175.33 versus £151.44, difference £23.89) and for antidepressant drugs (£70.11 versus £20.79, difference £49.32). The total average extra cost of the intervention was therefore £334.86 (95% confidence interval £276 to £393) per patient over 6 months (…)*.”

## Discussion

CReDECI 2 is a reporting guideline with 13 items, which offers guidance for a comprehensive reporting of the development, piloting, and evaluation of complex interventions in healthcare. The reporting guideline has been revised based on a formal consensus process, following the recommendations of the EQUATOR network [18]. The attendees of the consensus conference were scientists from several European countries with methodological and clinical expertise in the development and evaluation of complex interventions, partly also with experience as journal editors. This composition of the consensus group is comparable to other reporting guidelines [31] and allowed the integration of a broad range of competencies and experiences. The number of attendees, 45, of the consensus conference was in the upper range of other reporting guidelines [31].

Complex interventions are an important topic in healthcare research and there are several frameworks available offering methodological guidance [32]. In all frameworks, the use of qualitative and quantitative study designs is recommended during the research process of developing and evaluating complex interventions [4,33]. Available reporting guidelines, which include items relevant for complex interventions, focus on the evaluation stage (Table 1). In contrast, CReDECI 2 covers all relevant methodological aspects that should be reported during the research process of the development, piloting, and evaluation of a complex intervention, but without focusing on specific study designs. Although a report of the main characteristics of the intervention and its development should be included in the publication of the evaluation trial, an additional publication on the development and piloting of the intervention is often needed to report all relevant information.

To ensure comprehensive reporting of all study design-specific criteria, further available reporting guidelines should be used [3].

Like the original criteria list [7], the CReDECI 2 criteria are organized according to the first three stages of the MRC framework for the development and evaluation of complex interventions [4]. However, the guideline was developed based on relevant methodological literature on complex interventions, without focusing on a specific framework. Therefore, CReDECI 2 can be used for all complex interventions, irrespective of the methodological framework or model guiding its development and evaluation. The last stage of the MRC framework [4], long-term implementation, is not part of CReDECI 2, which focuses on the development and evolution of the interventions, whereas the fourth stage targets interventions which have proved to be effective [7].

The reporting quality of complex interventions varied in different analyses and in different stages. Several studies showed that reporting of the development and evaluation of complex interventions is insufficient [34-36]. There is evidence that the endorsement of CONSORT by healthcare journals has led to an improvement in reporting quality [37]. Although, evidence is lacking for other reporting guidelines [38], it seems likely that applying CReDECI 2 will lead to an improved reporting quality in complex interventions. CReDECI is listed in the overview of the EQUATOR network [3] and used in the Agency for Healthcare Research and Quality’s research white paper on systematic reviews of complex interventions [39].

## Conclusions

With CReDECI 2, a formally consented revised reporting guideline becomes available, which guides manuscript preparation in the development, piloting, and evaluation of complex interventions. The original guideline showed its practicability [17] and since the structure of CReDECI 2 remains unchanged, this seems valid for the revised guideline too.

Although CReDECI aims to cover most of the processes of developing and evaluating complex interventions, it is relatively concise as, for example, it does not focus on design-specific methodological details. Therefore, CReDECI 2 could be used as a specific instrument for complex interventions alongside design-specific reporting guidelines, for example, CONSORT for RCTs [8]. The decision on the choice of additional design-specific instruments has to be left to the discretion of study authors or reviewers.

## Additional files

Additional file 1:
**Changes from the original CReDECI to the revised CReDECI 2 criteria list.**
Additional file 2:
**REFLECTION CReDECI Consensus Group, list of participants.**

## Abbreviations

CReDECI: Criteria for Reporting the Development and Evaluation of Complex Interventions in Healthcare
MRC: British Medical Research Council
RCT: Randomized controlled trial
