# Supplementary material for: Criteria for Reporting the Development and Evaluation of Complex Interventions in healthcare: revised guideline (CReDECI 2)
Source: Trials. 2015 May 3;16:204. doi: 10.1186/s13063-015-0709-y (PMC4461976; doi:10.1186/s13063-015-0709-y)
Supplement: Additional file 1: — Changes from the original CReDECI to the revised CReDECI 2 criteria list. [file 13063_2015_709_MOESM1_ESM.pdf]

Additional file 1 – Changes from the original CReDECI to the revised CReDECI 2 criteria list

**Original CReDECI** [7]	**CReDECI 2**
First stage - Development	***First stage - Development***
1. Description of the intervention's underlying theoretical considerations	1. Description of the intervention's underlying theoretical basis
2. Description of all components of the intervention	2. Description of all intervention components, including the reasons for their selection as well as their aims/essential functions
3. Rationale for the selection of the intervention's components	
5. Rationale for the aim/essential functions of the intervention's components, including the evidence whether the components are appropriate for achieving this goal	
4. Illustration of any intended interactions between different components	3. Illustration of any intended interactions between different components
6. Consideration of contextual factors and determinants of the setting in the modelling of the intervention	4. Description and consideration of the context's characteristics in intervention modelling
Second stage - Feasibility and piloting	***Second stage - Feasibility and piloting***
7. Information on pilot-testing	5. Description of the pilot-test and its impact on the definite intervention
8. In case of pilot-test: Presentation of all relevant results and their impact on the modelling of the final intervention	
**Third stage - Introduction of the intervention and evaluation**	**Third stage - Evaluation**
9. Description of the control intervention (comparator)	6. Description of the control condition (comparator) and reasons for the selection
10. If the study was conducted in different clusters or centres: Description of a standardised implementation strategy throughout the centres	7. Description of the strategy for delivering the intervention within the study context
11. Description of all materials/ tools used for	8. Description of all materials or tools used

the implementation of the intervention to allow a replication of the study	for the delivery of the intervention
13. Description of any deviation from the study protocol during the implementation process	9. Description of fidelity of the delivery process compared to the study protocol
12. Description of an evaluation of the implementation process	10. Description of a process evaluation and its underlying theoretical basis
14. Description of facilitators or barriers revealed by the process evaluation which have influenced the interventions' implementation	11. Description of internal facilitators and barriers potentially influencing the delivery of the intervention as revealed by the process evaluation
	12. Description of external conditions or factors occurring during the study which might have influenced the delivery of the intervention and/or mode of action (i.e. how it works)
15. Description of unexpected interactions between components of the intervention and the environment in which the intervention was implemented	-
16. Description of costs or required resources for the intervention's implementation	13. Description of costs or required resources for the delivery of the intervention
