# Supplementary material for: Criteria for Reporting the Development and Evaluation of Complex Interventions in healthcare: revised guideline (CReDECI 2)
Source: Trials. 2015 May 3;16:204. doi: 10.1186/s13063-015-0709-y (PMC4461976; doi:10.1186/s13063-015-0709-y)
Supplement: Additional file 2: — REFLECTION CReDECI Consensus Group, list of participants. [file 13063_2015_709_MOESM2_ESM.pdf]

Additional file 2 - REFLECTION CReDECI Consensus Group

In addition to the authors, the following researchers supported the CReDECI consensus project (by completing the online feedback questionnaire and/or attending the consensus conference):

Mahmoud Al Kalaldeh, Zarqa University, Zarqa, Jordan; Odete Araújo, University of Minho, Braga, Portugal; Katrin Balzer, University of Lübeck, Lübeck, Germany; Sivera Berben, Radboud University Nijmegen, Nijmegen, The Netherlands; Nienke Bleijenberg, University Medical Center Utrecht, Utrecht, The Netherlands; Gunilla Borglin, Malmö University, Malmö, Sweden; Caroline Bradbury-Jones, University of Manchester, Manchester, United Kingdom; Sarah Byford, King´s College London, London, United Kingdom; Ricardo Cadima, Academy for Health Care Professions, Mönchengladbach, Germany; Gunilla Carlsson, Lund University, Lund, Sweden; Anna Castaldo, Provincia Religiosa S. Marziano di don Orione – Piccolo Cottolengo, Milan Italy; Sophie Cès, Université Catholique de Louvain, Louvain-la-Neuve, Belgium; Martin Dichter, Deutsches Zentrum für Neurodegenerative Erkrankungen, Witten, Germany; Margita Drienovsk, Faculty Hospital Nitra, Nitra, Slovakia; Lisa Ekstam, Lund University, Lund, Sweden; Agneta Malmgren Fänge, Lund University, Lund, Sweden; Andrea Giordano Foundation IRCCS Neurological Institute C. Besta, Milan, Italy; Unto Häkkinen, University of Exeter, Exeter, United Kingdom; Ruth Harris, Kingston University and St. George's University of London, London, United Kingdom; Tove Aminda Hanssen, University Hospital North-Norway, Tromsø, Norway; Henna Hasson, Karolinska Institutet, Stockholm, Sweden; Maria Adriana Pereira Henriques, University of Lisbon, Lisbon, Portugal; Oliver Rudolf Herber, Heinrich-Heine-University, Düsseldorf, Germany; Jacqueline Hill, University of Exeter, Exeter, United Kingdom; Daniela Holle, Deutsches Zentrum für Neurodegenerative Erkrankungen, Witten, Germany; Breeda Howley, University College Dublin, Dublin, Ireland; Natalja Istomina, Klaipeda University, Klaipeda, Lithuania; Lisa Jeffers, Northern Ireland Regional Genetics Service, Belfast, United Kingdom; Helena José, Catholic University of Portugal, Lisbon, Portugal; Marlène Karam, Catholic University of Louvain, Louvain-la-Neuve, Belgium; Susanne Kean, School of Health in Social Science, Edinburgh, United Kingdom; Helena Leino-Kilpi, University of Turku, Turku, Finland; Mette Spliid Ludvigsen, Aarhus University Hospital, Aarhus, Denmark; Tone Elin Mekki, University College of Bergen, Bergen, Norway; Riitta Meretoja, University of Turku, Turku, Finland; Teresa Moreno-Casbas, Joanna Briggs Institute for Evidence Based Health Care, Madrid, Spain; Caroline Nicholson, King’s College London, London, United Kingdom;

Alvisa Palese, Udine University, Udine, Italy; Rebecca Palm, Deutsches Zentrum für neurodegenerative Erkrankungen, Witten, Germany; Ľuboslava Pavelová, Constantine The Philosopher University, Nitra, Slovakia; Katherine Payne, University of Manchester, Manchester, United Kingdom; Mona Kyndi Pedersen, Aalborg University Hospital, Aalborg, Denmark; Ľubica Poledníková, Constantine The Philosopher University, Nitra, Slovakia; David Richards, University of Exeter, Exeter, United Kingdom; Martina Romanová, Constantine the Philosopher University, Nitra, Slovakia; Rafaela Rosário, University of Minho, Braga, Portugal; Evanthia Sakellari, University of Turku, Turku, Finland; Mária Semanišinová, Constantine The Philosopher University, Nitra, Slovakia; Walter Sermeus, University of Leuven, Leuven, Belgium; Margaret Smith, Queen Margaret University, United Kingdom; Andrea Solgajová, Constantine The Philosopher University, Nitra, Slovakia; Venetia-Sofia Velonaki, University Hospital of Lausanne, Lausanne, Switzerland; Tomas Sollar, Constantine the Philosopher University, Nitra, Slovakia; Riitta Suhonen, University of Turku, Turku, Finland; Julie Taylor, University of Edinburgh, Edinburgh, United Kingdom; Jana Turzáková, Constantine the Philosopher University in Nitra, Slovakia; Styliani Tziaferi, University of Peloponnese, Sparti, Greece; Therese Van Durme, University of Louvain, Brussels, Belgium; Filipa Veludo, Catholic University of Portugal, Pinhal Novo, Portugal.
